# c-Jun N-terminal kinase 2 prevents luminal cell commitment in normal mammary glands and tumors by inhibiting *p53/Notch1* and *breast cancer gene 1* expression

**DOI:** 10.18632/oncotarget.3787

**Published:** 2015-04-25

**Authors:** Michael A. Cantrell, Nancy D. Ebelt, Adam D. Pfefferle, Charles M. Perou, Carla Lynn Van Den Berg

**Affiliations:** ^1^ Institute of Cellular & Molecular Biology, College of Pharmacy, University of Texas at Austin, Dell Pediatric Research Institute, Austin, TX 78723, USA; ^2^ Division of Pharmacology &Toxicology, College of Pharmacy, University of Texas at Austin, Dell Pediatric Research Institute, Austin, TX 78723, USA; ^3^ Department of Pathology and Laboratory Medicine, University of North Carolina at Chapel Hill, School of Medicine, Chapel Hill, NC 27599, USA; ^4^ Lineberger Comprehensive Cancer Center, University of North Carolina at Chapel Hill, School of Medicine, Chapel Hill, NC 27599, USA; ^5^ Department of Genetics, University of North Carolina at Chapel Hill, School of Medicine, Chapel Hill, NC 27599, USA

**Keywords:** breast cancer, JNK2, p53, Notch1, BRCA1

## Abstract

Breast cancer is a heterogeneous disease with several subtypes carrying unique prognoses. Patients with differentiated luminal tumors experience better outcomes, while effective treatments are unavailable for poorly differentiated tumors, including the basal-like subtype. Mechanisms governing mammary tumor subtype generation could prove critical to developing better treatments. C-Jun N-terminal kinase 2 (JNK2) is important in mammary tumorigenesis and tumor progression. Using a variety of mouse models, human breast cancer cell lines and tumor expression data, studies herein support that JNK2 inhibits cell differentiation in normal and cancer-derived mammary cells. JNK2 prevents precocious pubertal mammary development and inhibits Notch-dependent expansion of luminal cell populations. Likewise, JNK2 suppresses luminal populations in a p53-competent Polyoma Middle T-antigen tumor model where *jnk2* knockout causes p53-dependent upregulation of *Notch1* transcription. In a *p53* knockout model, JNK2 restricts luminal populations independently of Notch1, by suppressing *Brca1* expression and promoting epithelial to mesenchymal transition. JNK2 also inhibits estrogen receptor (ER) expression and confers resistance to fulvestrant, an ER inhibitor, while stimulating tumor progression. These data suggest that therapies inhibiting JNK2 in breast cancer may promote tumor differentiation, improve endocrine therapy response, and inhibit metastasis.

## INTRODUCTION

In the pubertal mammary gland, ductal branches invade and extend into the fat pad guided by transient bulbous structures called terminal end buds (TEBs). Mammary stem and progenitor cells reside in TEBs while differentiated, apical luminal cells and basally located myoepithelial cells form the duct as extension proceeds. During this process, mammary stem cells respond to stimuli that facilitate commitment into luminal or basal lineages. Current evidence suggests that mammary stem and/or luminal progenitor cells are the probable targets for breast cancer development [1–3]. Thus, it is important to interrogate these populations to understand how cell differentiation affects susceptibility to transformation or tumor progression. Insight into integral differentiation pathways may provide opportunities for new treatments with the goal of targeting tumor initiating cells, which having undergone epithelial to mesenchymal transition (EMT), promote treatment resistance, tumor recurrence and metastasis.

Multiple pathways contribute to mammary lineage commitment in both normal and tumor cells. Of the four Notch receptors, Notch1 shows the highest abundance in luminal cells [4]. Notch1 enhances luminal populations [4–6] whereas, its counterpart ΔNp63 is highly expressed in basal populations. In the normal mammary gland, Notch1 and ΔNp63 regulate each other's expression in opposing fashions to sustain a homeostatic balance between luminal and myoepithelial cell populations. In breast tumors, persistent Notch1 activity in tumor initiating or progenitor cells may have a negative impact on tumor progression [7, 8].

Similar to Notch1, BRCA1 is critical for differentiation of progenitors into ER^+^ luminal cells [9]. BRCA1 induces ER transcription [10] and inhibits transcriptional machinery associated with de-differentiation and EMT [1, 11]. A high proportion of mutant BRCA1 breast tumors are classified as basal-like, [12–14], likely because mutations in BRCA1 block terminal differentiation and cause luminal progenitor expansion. Experimental transformation of these progenitors generates tumors that closely reflect those of BRCA1 mutation carriers with basal-like features [1–3]. Aside from hereditary breast cancer, low BRCA1 expression occurs in sporadic breast cancers by means of promoter methylation or overexpression of transcriptional repressors [15, 16]. These studies suggest that a reduction in wildtype protein inhibits mammary cell differentiation. While recognized as a critical player in breast cancer risk and behavior, no approaches have been developed to enhance BRCA1 function in breast cancer to improve patient outcome. Correspondingly, therapeutically targeting basal and tumor initiating cells with EMT properties is challenging due to their low proliferative index. These findings emphasize the need to identify “druggable” targets that control mammary cell differentiation.

JNK1 and JNK2 isoforms are ubiquitously expressed. Through phosphorylation of various substrates, most notably c-Jun, JNKs govern apoptosis, proliferation, motility, and differentiation. Knockout or knockdown of JNK2 inhibits mammary tumor cell migration and invasion along with tumor growth and metastasis in response to receptor tyrosine kinases [17, 18]. High JNK2 expression in human basal-type tumors is also associated with shorter disease free survival [17]. These key results motivated us to explore novel roles of JNK2 in normal and cancer related cell lineage commitment.

Using *jnk2* knockout (*jnk2ko)* mice and cell lines, we develop a model by which JNK2 inhibits luminal differentiation in normal and cancerous mammary epithelial cells through two mechanisms that depend on p53 status. In p53 competent cells, JNK2 lowers p53 expression, and consequently Notch1 expression, to limit luminal populations. In the absence of p53, JNK2 prevents luminal differentiation by inhibiting BRCA1 and ER expression. Through these diverse means, it serves a central role in mammary cell lineage commitment and enhances tumor initiating cells and metastasis. These results suggest that targeting JNK2 in breast tumors may expand the population of therapy sensitive cells and consequently improve patient outcomes.

## RESULTS

### Jnk2 loss causes precocious mammary development and alters mammary epithelial cell differentiation

**Figure 1:**
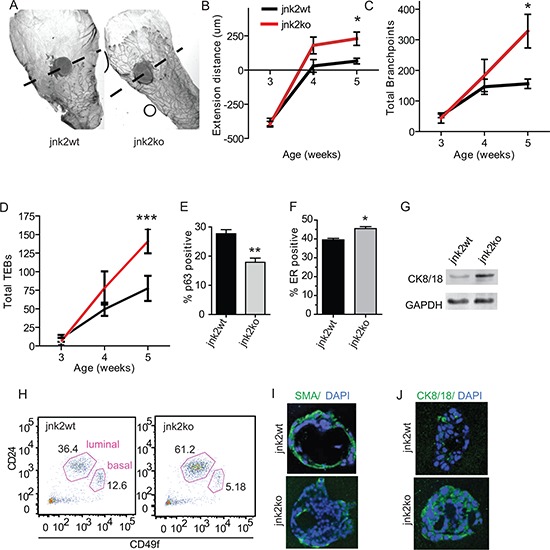
Absence of JNK2 accelerates pubertal mammary development and alters mammary cell differentiation **A.** Representative whole mounts of mammary glands from *jnk2wt* and *jnk2ko* mice at puberty (5 wk-old); **B-D.** Quantification of ductal extension, branching, and total terminal end buds from pre-puberty (3wk-old) and puberty (*n* = 5); **E-F.** Quantification of p63^+^ basal cells and ER^+^ luminal cells in adult ducts (*n* = 3); **G.** Western blot of CK8/18 expression in adult mammary organoids; **H.** Representative CD24 and CD49f staining of adult mammary cells; **I-J.** Representative images of Smooth Muscle Actin (SMA)^+^ and CK8/18^+^ in 3D cultures. Nonparametric *t*-tests were performed for all analyses except Linear Regression analysis was used for D. *p < 0.05, **p < 0.001, ***p < 0.0001.

To investigate if JNK2 affects mammary development, glands were harvested from female *jnk2wt* and *jnk2ko* virgin mice. By five weeks of age, ductal development of whole-mounted *jnk2ko* pubertal glands appear more advanced than *jnk2wt* glands as evidenced by ductal extension (Fig 1A and 1B, *p* = 0.012), increased secondary branching (Fig 1C, *p* = 0.0169), and increased number of TEBs (Fig 1D, *p* < 0.0001). By the end of puberty, glands of both genotypes completely fill the fat pad. These quantifications confirm that *jnk2ko* glands exhibit precocious pubertal development.

Analysis of adult *jnk2wt* glands shows that JNK2 is widely expressed in mammary epithelial cells (Fig 1SA). When staining for cell lineage markers, *jnk2ko* glands possess 35% fewer p63^+^ basal/myoepithelial cells than *jnk2wt* (Fig 1E, *p* = 0.0078 and Fig 1SB) with a reciprocal increase in ER^+^ cells (Fig 1F, *p* = 0.011 and Fig 1SC). Higher cytokeratin (CK)8/18 expression in *jnk2ko* organoids is shown by western blot (Fig 1G). To better quantify the luminal and basal cell populations, cell surface markers CD49f and CD24 were measured using flow cytometry. *Jnk2ko* glands contain 61% lin^−^/CD49f^Lo^/CD24^+^ luminal cells compared to 36% in *jnk2wt* glands (Fig 1H). This corresponds to a smaller basal population in the *jnk2ko* mammary epithelial cells.

Given that *jnk2* encodes a ubiquitously expressed protein and its deletion may lead to hormone-dependent alterations in mammary cell differentiation, we explored whether it could function cell autonomously in 3D organoid culture. Consistent with *in vivo* observations, the resulting *jnk2ko* acini show fewer smooth muscle actin (SMA)^+^ basal cells and more CK8/18^+^ luminal cells compared to the *jnk2wt* controls (Fig 1I, 1J). Moreover, the average acinar diameter is greatly enhanced in *jnk2ko* group (Fig S1D, *p* < 0.0001). While proliferation did not significantly differ (Fig S1E, *p* = NS), apoptosis indices did as evidenced by cleaved caspase 3 (Fig S1F, *p* = 0.0009), perhaps a consequence of precocious hollowing of *jnk2ko* acini. Together, these data support that JNK2 alters basal and luminal proportions in a cell autonomous fashion.

### JNK2 inhibits normal luminal mammary differentiation in a Notch1-dependent fashion

**Figure 2:**
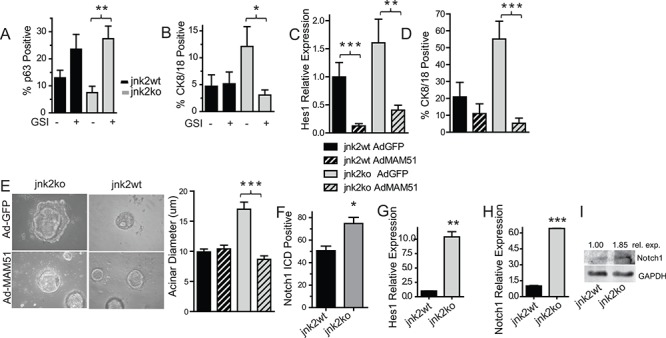
Elevated Notch1 in jnk2ko glands and mammary cells increases luminal cell populations **A-C.** 3D cultures were grown in the presence of Gamma Secretase Inhibitor (GSI) or DMSO and probed for p63^+^ basal A or CK8/18^+^ luminal cells B; C Isolated mammary cells in suspension were infected with adenoviral vectors encoding GFP (AdGFP) or AdMAM51 and grown in 3D culture. Hes1 expression was measured by qPCR in harvested cultures; **D.** CK8/18+ luminal cells were assessed by immunofluorescence in 3D cultures; **E.** Acinar diameter of 3D cultures was determined; **G.** Pubertal glands were probed for Notch1-ICD and analyzed within terminal end buds; **F-H.** Organoid RNA was analyzed for Hes1 G and Notch1 H expression using qPCR; **I.** Western blot of Notch1 protein in organoid samples. 1 Way ANOVA with post-hoc *t*-test was performed. Post-hoc nonparametric *t*-test was also performed for *p < 0.05, **p < 0.001, ***p < 0.0001.

Differentiation and development of the mammary gland is highly Notch-dependent [4–6, 19]. To determine if Notch promotes proliferation and/or differentiation of *jnk2ko* mammary epithelial cells, 3D organoid cultures were treated with Gamma Secretase Inhibitor (GSI) IX, a pan inhibitor of Notch cleavage/activation. After 11 days of GSI treatment, the proportion of p63^+^ basal cells in *jnk2ko* cultures significantly increases (Fig 2A and Fig S2A, *p* = 0.0006), and CK8/18+ populations decrease compared to vehicle controls (Fig 2B, *p* = 0.0092 and Fig S2B), suggesting a greater dependence on Notch signaling for luminal commitment compared to *jnk2wt* controls where no significant changes were observed.

These findings were validated by inhibiting Notch-dependent transcription with a dominant negative AdMAM51 [20] in 3D organoid culture. In this experiment, mammary epithelial cells were isolated and infected with AdGFP (Adenoviral GFP) or AdMAM51 then placed in culture. RNA later harvested from cultures show AdMAM51 reduces expression of *Hes1* in both genotypes (Fig 2C). Similar to GSI, AdMAM51 expression in *jnk2ko* mammary cells significantly decreases luminal CK8/18^+^ cell populations of *jnk2ko* cultures without significantly affecting populations in *jnk2wt* cultures (Fig 2D, *p* = 0.0111 and Fig 2SC). AdMAM51 also significantly reduces acinar diameter only in the *jnk2ko* cells (Fig 2E, *p* < 0.0001).

Notch reporter transgenic mice reveal that endogenous Notch activity is high in the pubertal gland [8]. Notch signaling was assessed by Notch1 intracellular domain (Notch1^ICD^) immunohistochemistry of mammary glands from mice in puberty. A significantly higher proportion of TEB cells stain positive in *jnk2ko* glands as compared to *jnk2wt* controls (Fig 2F, *p* = 0.0159 and Fig 2SD), but no differences in Notch1^ICD^ expression were detected in mature ducts. Localization of high Notch1^ICD^ is important because it allows greatest influence over differentiation and is also consistent with reported endogenous Notch activity being highest in TEBs [8].

To test if increased Notch1^ICD^ staining in *jnk2ko* pubertal glands leads to higher Notch-dependent transcription, *Hes1* expression was measured, using qPCR. *Jnk2ko* organoids express 10.5 times more *Hes1* mRNA than *jnk2wt* (Fig 2G, *p* = 0.0093). *Notch-1* mRNA is 6.4 times higher in *jnk2ko* organoids (Fig 2H, *p* < 0.0001), along with an increase in full-length Notch-1 protein as compared to *jnk2wt* (Fig 2I). Collectively, these data show that *jnk2ko* mammary epithelial cells are more sensitive to Notch signaling, and JNK2 regulates mammary epithelial cell differentiation via the Notch pathway.

### Jnk2 loss increases the proportion of luminal cells in polyoma middle T antigen (MT) mammary tumors in a p53/Notch1-dependent fashion

**Figure 3:**
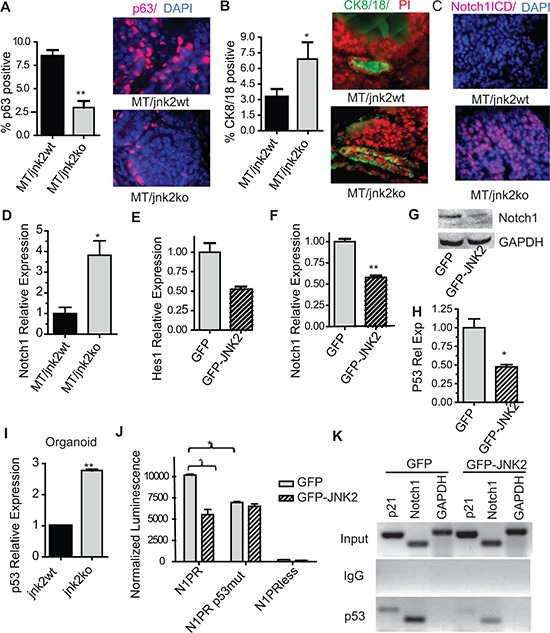
Absence of jnk2 in MT tumors increases luminal cell populations and enhances p53 and Notch1 expression **A-B.** Representative images and quantification of p63^+^ A or CK8/18^+^ B cells in MT tumors (*n* = 5 per genotype); **C.** Representative images of Notch1^ICD+^ cells in MT tumors; **D.** Notch1 expression by qPCR in MT tumors; **E-F.** *Hes1* E and *Notch1* **F** expression by qPCR in MT/*jnk2ko* cells; **G.** Western blot of full-length Notch1 in MT/*jnk2ko* GFP and GFP-JNK2 cells; **H.** p53 expression in MT/*jnk2ko* GFP and GFP-JNK2 cells by qPCR; **I.** *p53* expression in normal mammary organoids by qPCR; **J.** MT*/jnk2ko* cells were transfected with Notch1 promoter constructs and analyzed for promoter activity (N1PR= wildtype *Notch1* promoter, N1PRp53mut = *Notch1* promoter with mutated p53 response elements, N1PRless = promoter-less control plasmid); **K.** Chromatin Immunoprecipitation assay of p53 binding to p53 response elements within the p21 and Notch1 promoters in MT/*jnk2ko* cells. Gapdh promoter used as negative control. 1 Way ANOVA with post-hoc *t*-test was performed for **J** followed by post-hoc *t*-test. Nonparametric *t*-test was performed for all others, *p < 0.05, **p < 0.001.

The importance of decisive differentiation pathways in the normal gland in tumor differentiation is illustrated by the finding that basal-type tumors may originate from luminal progenitors rather than basal/myoepithelial cells [2]. To explore whether JNK2 affects tumor cell differentiation, we used an MT transgenic mouse model. MT tumors most closely reflect the phenotypic characteristics of human luminal breast cancer [21], [22]. MT/*jnk2wt* and MT/*jnk2ko* mammary tumors were immunostained with p63 or CK8/18 antibodies to identify basal and luminal cells, respectively. Although a high proportion of tumor cells do not express either marker, MT/*jnk2ko* tumors have significantly fewer p63^+^ nuclei and more CK8/18^+^ cells (Fig 3A and 3B, *p* = 0.0079 and *p* = 0.0411 respectively). Moreover, MT/*jnk2ko* tumors express Notch1^ICD^ in almost all cells, whereas MT/*jnk2wt* tumors are nearly void of its expression, (Fig 3C). Correspondingly, MT*/jnk2ko* tumors express four-fold higher *Notch1* mRNA (Fig 3D, *p* = 0.0101). These results suggest that, akin to the normal mammary gland, JNK2 inhibits luminal cell populations in the spontaneous MT tumor model.

To explore the mode by which JNK2 inhibits Notch1 expression and activity, we used a MT/*jnk2ko* cell line expressing GFP*-*JNK2 or GFP alone [23]. Consistent with JNK2 suppression of Notch1 signaling, *Hes1* mRNA is reduced GFP-JNK2 cells (Fig 3E, *p* = NS). Additionally, MT/*jnk2ko* GFP-JNK2 cells express less *Notch-1* mRNA (*p* = 0.0005) and protein than MT*/*jnk2ko GFP cells (Fig 3F and 3G).

p53 potentiates *Notch-1* transcription by binding to two p53 responses elements (REs) within its promoter [24]. Because MT tumors and cell lines express wildtype p53 [23], we hypothesized that JNK2 inhibits Notch1 in a p53-dependent fashion. This hypothesis is supported by the lower expression levels of *p53* mRNA in MT/*jnk2ko* cells expressing GFP-JNK2 (Fig 3H) and the presence of nearly three times more p53 mRNA in *jnk2ko* mammary organoids compared to *jnk2wt* (Fig 3I, *p* = 0.0004).

A potential relationship between JNK2 and p53 in regulating Notch1 expression was then tested. MT/*jnk2ko* GFP and GFP-JNK2 cells were transfected with luciferase plasmids containing either full-length Notch1 promoter (N1PR), Notch1 promoter with mutated p53REs (N1PRmut), or promoterless (N1PRless), as previously described [24]. As expected, JNK2 expression reduces N1PR activity (Fig 3J, *p* = 0.0170). Further, only the GFP cells show reduced N1PRmut promoter activity compared to N1PR, supporting a mechanism whereby JNK2 inhibits *Notch1* promoter activity by decreasing p53 expression or promoter binding.

To test if lower *p53* expression in MT/*jnk2ko* GFP-JNK2 cells leads to decreased p53 binding to the *Notch1* promoter, chromatin immunoprecipitation was performed. The *p21* promoter was a positive control for p53 binding. The GAPDH promoter was a negative control. Similar input is seen for all samples, and IgG antibody control does not amplify (Fig 3K). *Notch1* promoter primers show that more p53 binds to the promoter in MT/*jnk2ko* GFP cells than GFP-JNK2 cells, thus demonstrating that JNK2 dampens p53 binding to the *Notch1* promoter. Experiments above support that less p53 binding to the *Notch1* promoter in the presence of GFP-JNK2 occurs due to lower *p53* expression.

### Jnk2 loss in a p53ko model promotes tumor growth and luminal differentiation by augmenting BRCA1

**Figure 4:**
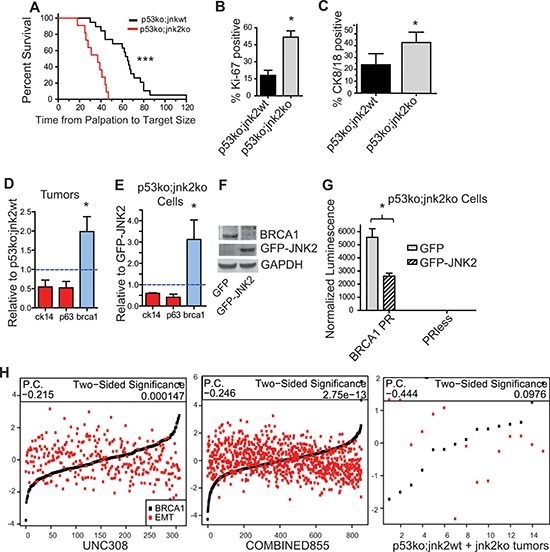
Absence of jnk2 increases the luminal cell population and BRCA1 expression in p53ko tumors **A.** *p53ko* tumors were measured until reaching 1.5 cm diameter (*n* = 22 *p53ko;jnk2wt*, *n* = 18 *p53ko;jnk2ko*, Log rank test); **B-C.** *p53ko* tumors were immunostained and Ki-67^+^ and CK8/18^+^ cells were quantified (*n* = 5); **D-E.** Expression of basal (red) and luminal (blue) markers was measured in *p53ko* tumors (*n* = 8, E) and *p53ko* cell lines F. Western blot of BRCA1 expression in *p53ko* cells; **G.** *p53ko* cells were transfected with *Brca1* promoter (BRCA1PR) or promoterless control (PRless) luciferase plasmids and assayed for promoter activity; **H.** Correlation of Brca1 expression and EMT-related gene expression was assessed in human tumors (UNC308, *n* = 308, and COMBINED855, *n* = 855) and *p53ko* mouse tumors (*n* = 15, P.C. = Pearson Correlation). A nonparametric, two-tailed *t*-test was used to detect statistical differences between two groups. The Pearson's correlation was performed using data in H. *p < 0.05, **p < 0.001, ***p < 0.0001.

We next performed mammary gland transplants of *p53ko;jnk2wt* and *p53ko;jnk2ko* tissue into *wt* mice to assess whether JNK2 controls tumor development or mammary cell lineage commitment independently of p53 [25]. Similar to reports comparing *p53ko;jnk2wt* and *p53ko;jnk2ko* systemic mutant mice [26], no difference in tumor latency was noted. However, *p53ko;jnk2ko* tumors grow significantly faster than *p53ko;jnk2wt* tumors after palpation (Fig 4A, *p* < 0.0001). *p53ko;jnk2ko* tumors also exhibit 2.9 times more Ki-67 positive cells compared to *p53ko;jnk2wt* (Fig 4B, *p* = 0.0159). The JNK2 anti-proliferative response seen with the *p53ko* model differs from that observed in the MT model expressing wildtype p53 [23]. These data indicate that JNK2 may influence tumor growth through various downstream targets including p53.

While others have observed that Notch1 increases cell proliferation, its expression is not statistically different in the *p53ko* tumors by microarray analysis (1.1-fold, FDR 54%) or qPCR of tumors (Fig S3A, *p* = NS). Because these results may reflect the heterogeneity of the model, Notch1 promoter activity was measured in *p53ko* primary tumor cells. GFP-JNK2 or GFP were stably expressed in a *p53ko;jnk2ko* cell line. *Notch1* promoter activity is not changed by JNK2 in *p53ko* cells (Fig S3B, *p* = NS), supporting that p53 is required for JNK2 to reduce *Notch1* transcription.

### Absence of jnk2 augments CK8/18+ populations and BRCA1 expression

As with MT tumors, a large proportion of cells within tumors do not stain positive for differentiation markers, but the proportion of CK8/18^+^ cells is significantly higher in *p53ko;jnk2ko* tumors compared to *p53ko;jnk2wt* (Fig 4C, *p* = 0.0159), akin to the normal mammary gland and MT tumors. Further, while expression of basal related genes, p63 and CK14, does not significantly differ, the luminal marker *Brca1* is upregulated in the *p53ko;jnk2ko* tumors (Fig 4D, *p* = 0.05). BRCA1 expression also differs by tumor microarray (BRCA1 elevated 2.16-fold, 4.38% FDR) along with alterations in the ATM/BRCA1 pathway using GSEA analysis (Fig S3C, S3D). The *p53ko;jnk2ko* cell line was used to test if JNK2 alters lineage associated genes. Fig 4E shows that the GFP cells have significantly higher *Brca1* mRNA compared to GFP-JNK2 cells (*p* = 0.0205). BRCA1 protein is also elevated in GFP cells (Fig 4F). To confirm that BRCA1 transcription is inhibited by JNK2, *p53ko;jnk2ko* GFP and GFP-JNK2 cells were transfected with a BRCA1 promoter plasmid or promoterless plasmid. GFP-JNK2 reduces BRCA1 promoter activity compared to the GFP control (Fig 4G, *p* = 0.0127), supporting that BRCA1 is a JNK2 target in the absence of p53. *Brca1* expression did not differ by qPCR in MT tumors (data not shown), further suggesting that *brca1* is a JNK2 target only in the absence of p53. These data show that JNK2 inhibits luminal lineage commitment independently of p53.

To explore the mechanism of luminal cell suppression by JNK2, we considered other known mechanisms of differentiation in the normal mammary gland. BRCA1 controls the latter stages of luminal differentiation and its low expression is associated with EMT and stem cell populations. We used these gene signatures to test whether JNK2 prevents tumor cell differentiation. First, we examined if BRCA1 expression is negatively correlated with an EMT signature in human tumors. For this analysis, the average value of genes increasing in expression during EMT was calculated [27], then the correlation between EMT and BRCA1 expression was plotted in our mouse and human datasets. Fig 4H shows a negative correlation between BRCA1 and EMT in both the UNC308 [28] (Pearson correlation −0.215, *p* = 0.000147) and COMBINED855 [29] (Pearson correlation −0.246, *p* = 2.75e^−17^) human datasets. Only a trend was observed in the relatively smaller number *p53ko* mouse tumors (Pearson correlation −0.444, *p* = NS). These data are in agreement with BRCA1 enhancing luminal differentiation.

Expression differences of select EMT-related genes were then explored by comparing *p53ko;jnk2ko* to *p53ko;jnk2wt* mouse tumors and the *p53ko;jnk2ko* GFP to GFP-JNK2 cell lines. Expression levels are heterogeneous, but *p53ko;jnk2ko* tumors show trends toward lower expression of mesenchymal transcription factors (*Twist1, Snai1, Klf4*, *Zeb1*, and *Snai2*) and significantly elevated expression of *Cdh1*, the gene encoding e-cadherin (Fig S4A). Microarray analysis of the GFP and GFP-JNK2 cell lines identified several EMT-related genes that were confirmed by western blot (Fig S4B, S4C). The effect of JNK2 on JNK1 expression was also assessed by Western blot, showing that GFP-JNK2 only slightly increases JNK1 expression (Fig S4D). These data support that downregulation of *Brca1* by JNK2 is associated with EMT in the *p53ko* model and that a compensatory increase JNK1 expression is not observed in the absence of *jnk2*.

### BRCA1 is sufficient to reduce JNK2 enriched tumor initiating cells

In spite of differences in EMT gene expression and luminal cell markers, a *p53ko;jnk2wt* and *p53ko;jnk2ko* tumor microarray failed to show a significant alteration of differentiation scores on a transcriptome level [28]. We postulate that this may be due to host (*p53wt;jnk2wt*) stromal cells infiltrating the tumors, the high heterogeneity of *p53ko* tumor model [30], or the likelihood that some genes are distally regulated by JNK2. Thus, we focused on a *p53ko;jnk2ko* cell line for subsequent studies in tumor cell differentiation.

**Figure 5:**
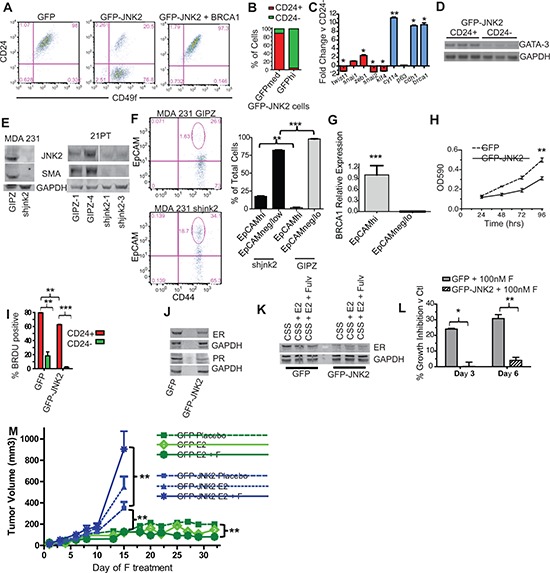
JNK2 increases putative tumor initiating cells and inhibits ER expression in p53ko mouse and mutant p53-expressing human cells **A.** CD24/CD49f staining was compared in *p53ko* cell lines by flow cytometry; **B.** The percentage of CD24^−^ and CD24^+^ cells were assessed in *p53ko;jnk2ko* GFP-JNK2 cells that were gated for GFP expression (medium and high) by flow cytometry; **C.** CD24^+^ and CD24^−^ populations in *p53ko;jnk2ko* GFP-JNK2 cells were separated by FACS and expression of EMT/stem (red) and differentiation (blue) markers measured by qPCR; **D.** CD24^+^ and CD24*-* populations in *p53ko;jnk2ko* GFP-JNK2 cells were tested for Gata-3 expression by RT-PCR; **E.** shJNK2 or GIPZ non-silencing plasmids were stably expressed in mutant p53-expressing MDA 231 and 21PT cell lines. JNK2 and SMA expression were measured by western blot; **F.** MDA 231 cells were assessed for EpCAM and CD44 expression; **G.** EpCAM^hi^ and EpCAM^neg/lo^ populations in MDA 231 cells were separated by FACS. *Brca1* was measured by qPCR; **H.** Cell viability of *p53ko* cells was evaluated using MTT assay; **I.** *p53ko* cells were pulse labeled with BrdU. BrdU incorporation in CD24*^+^* and CD24*^−^* populations was measured; **J.** Western blot of ER and PR expression in *p53ko* cells; **K.** *p53ko* cells were cultured with charcoal stripped serum (CSS), CSS + Estradiol (E2), or CSS + E2 + Fulvestrant (F) and ER expression was measured by western blot; **L.** *p53ko* cells were cultured in full medium with or without F. Cell viability was measured at times indicated. Suppression of growth is shown as percentage of DMSO control growth for each genotype; **M.** Effect of E2 and E2-F treatment on orthotopically growing *p53ko;jnk2ko* GFP and GFP-JNK2 cells.

Thus far our *p53ko* model indicates that JNK2 inhibits luminal cell populations by expanding the EMT-expressing tumor initiating cell population. To test this possibility, we measured CD24 and CD49f to quantify putative tumor initiating cells. The CD24^−/Lo^ population has been identified as an EMT/stem/tumor initiating cell population in mouse mammary tumor models. CD49f expression is high in mammary stem cells and tumor initiating cells [31, 32], whereas CD44^+^ cells fail to show increased tumor formation in a limiting dilution assay in this model [33]. While CD49f positivity does not differ between GFP and GFP-JNK2 cells, CD24 expression is markedly changed. GFP cells are 98% CD49f^+^/CD24^+^ cells and 1.4% CD49f^+^/CD24^−^ cells versus GFP-JNK2 cells (21% CD49f^+^/CD24^+^ and 77% CD49f^+^/CD24^−^) (Fig 5A, *p* < 0.0001). Stable-expression of BRCA1 in GFP-JNK2 cells shifts the CD24^−^ population from 77% to < 1% (Fig 5A, *p* < 0.0001), consistent with its inverse correlation with EMT shown in Fig 4I. Interestingly, when GFP-JNK2 cells are gated for medium and high GFP intensity, high JNK2 (GFP) expression occurs primarily in CD24^−^ cells (Fig 5B).

The CD24^+^ population is more differentiated than the CD24^−^ population as shown by less *Twist1* (*p* = 0.05)*, Klf4* (*p* < 0.0001), and *Snai2* (*p* = 0.0233) and higher *Brca1* (*p* = 0.05)*, Ck14* (*p* = 0.0018) and *Cdh1* expression (*p* = 0.0131, Fig 5C). *Gata-3* expression, a transcription factor expressed by luminal cells [34], was compared using RT-PCR. Its expression is also higher in the CD24^+^ population (Fig 5D, *p* = 0.0209). Using the GFP-JNK2 cells, two populations can be also visualized using vimentin and e-cadherin immunocytochemistry (Fig S5A). In bright field analysis, GFP-JNK2 cells are less contact inhibited than GFP cells, further supporting an EMT phenotype (Fig S5B). Similar relationships between JNK2 expression and EMT/differentiation are present in MDA-231 and 21PT human cell lines. Here, stable expression of shJNK2 significantly decreases expression of SMA, a myoepithelial marker (Fig 5E). shJNK2 expression in MDA-231 cells significantly increases EpCAM^hi^ epithelial cells and decreases EpCAM^neg/lo^ basal/mesenchymal populations (Fig 5F, *p* = 0.0013 and *p* < 0.0001). The EpCAM^hi^ population expresses significantly higher levels of *Brca1* than the EpCAM^neg/lo^ population (Fig 5G, *p* < 0.0001). Together, these data maintain that JNK2 suppresses BRCA1-expressing epithelial populations and promotes an EMT phenotype in mouse and human cells.

The functional significance of JNK2 induced EMT was then tested with a limiting dilution tumor formation assay using *p53ko;jnk2ko* GFP or GFP-JNK2 cells. All mice developed palpable tumors when injected with 10^4^ cells (Table 1). However, GFP masses remained relatively small, not reaching target size by 6 months. At 10^3^ cells, 1 of 4 GFP-JNK2 injected mice developed a palpable tumor, but no GFP cell injected mice did. At 10^2^ cells, no mice experienced palpable tumors in either group. When euthanizing tumor bearing mice, internal organs and mammary glands were inspected for GFP^+^ foci. No metastases were observed in GFP cell injected mice, but two metastases were detected in GFP-JNK2 cell injected mice.

**Table 1:** JNK2 promotes tumor growth and metastasis in p53ko cells Limiting dilution assay was performed in nude mice by injection of cell numbers shown. Palpated tumors were grown to harvest diameter of 1.5cm and metastases were assessed by fluorescence microscopy. Indolent tumors that were not detected by palpation were assessed by fluorescence microscopy.

Cell # injected	Palpated Tumors				Indolent Tumors			
	GFP		GFP-JNK2		GFP		GFP-JNK2	
	Tumors	Metastasis	Tumors	Metastasis	Tumors	Metastasis	Tumors	Metastasis
**10^4^**	4/4	0/4	4/4	2/4	0/0	0/0	0/0	0/0
**10^3^**	0/6	0/6	1/4	0/4	0/6	0/6	1/3	2/3*
**10^2^**	0/6	0/6	0/9	0/9	0/6	1/6	4/9	1/9

* One mouse that was injected with GFP-JNK2 cells developed ascites.

After 6 months of observation, some mice from each genotype remained tumor free. To explore if the injected cells were still viable but indolent, internal organs and mammary glands were examined for GFP. No foci were detected in GFP cell injected mice, but one contra-lateral gland metastasis was seen at 10^2^ cell dose without a primary tumor. Five tumors were found in GFP-JNK2 injected mice, with one at 10^3^ cells and four at 10^2^ cells. A contralateral gland metastasis was seen in each of the 10^3^ and 10^2^ cell dilutions and ascites developed in one of the 10^3^ cell GFP-JNK2 group. Data from this experiment point toward the GFP cell line having approximately 1 tumor initiating cell in 5,116 cells versus the GFP-JNK2 cell line having 1 in 569 cells (Table 2, *p* = 0.0305, Pearson's Chi-squared Test). This experiment shows that GFP-JNK2 promotes tumor initiating cell populations, which also possess a propensity to metastasize. These two properties likely result from JNK2 promoting EMT.

**Table 2:** JNK2 expression promotes tumor initiating cell populations in p53ko cells. Data from limiting dilution assay were used to calculate tumor initiating cell content in p53ko cell lines

	GFP	GFP-JNK2
**Frequency**	1 in 5116	1 in 569
− S.E.M.	1 in 8605	1 in 937
+ S.E.M.	1 in 3042	1 in 346

### JNK2 inhibits tumor cell proliferation

As noted earlier, *p53ko;jnk2ko* tumors grow faster than *p53ko;jnk2wt* controls. *In vitro* proliferation is also inhibited by GFP-JNK2 in *p53ko;jnk2ko* cells (Fig 5H, *p* = 0.0002). These findings are consistent with an EMT/tumor initiating cell population associated low proliferation index. To examine if the JNK2 effects on proliferation result from EMT, cells were pulse labeled with BrdU and sorted into CD24^−^ and CD24^+^ populations. CD24^+^ populations from both genotypes incorporate BrdU at a significantly higher frequency than CD24^−^ populations (Fig 5I, *p* = 0.0089 for GFP and *p* < 0.0001 for GFP-JNK2). Both the CD24^+^ and CD24^−^ GFP-JNK2 cells incorporate less BrdU compared to CD24^+^ and CD24^−^ GFP (*p* < 0.0001), indicating that JNK2 slows *in vitro* proliferation in both an EMT dependent and independent fashion in the *p53ko;jnk2ko* cell line.

### JNK2 reduces ER expression

In rationalizing how the CD24^+^ population displays a higher proliferation rate despite evidence of more luminal differentiation, we suspected that ER could modulate this response, in light of its elevated expression in GFP cells (Fig S4B). Indeed, GFP cells express more ER protein along with its target gene, progesterone receptor (PR) than GFP-JNK2 cells (Fig 5J). The functional importance of ER was determined by exposing cells to estradiol (E2) or E2+fulvestrant, a drug that degrades ER protein. After 24 hours, ER protein is lower in GFP cells treated with E2+fulvestrant, whereas E2+fulvestrant treatment of GFP-JNK2 cells shows no change in ER (Fig 5K). Moreover, fulvestrant significantly inhibits GFP cell viability on day 3 and 6 (*p* < 0.0023 and *p* = 0.0006, respectively), but it had no effect on GFP-JNK2 cells (Fig 5L). These data show that JNK2 inhibits ER expression and ER associated proliferation.

The potential clinical impact of JNK2 on ER dependent tumor growth was then explored. GFP and GFP-JNK2 cells were injected into ovariectomized *nu/nu* mice one day after inserting estradiol pellets. Placebo or fulvestrant treatment was started upon tumor palpation. Surprisingly, while GFP associated masses became palpable they remained small for the duration of the experiment (Fig 5M), irrespective of treatment. In contrast, all GFP-JNK2 tumors eventually reached ≥ 750 mm^3^. Placebo treated GFP-JNK2 tumors grew significantly more rapidly than the GFP placebo group (*p* = 0.0003, Day 15) but grew more slowly compared to the GFP-JNK2 tumors treated with E2+fulvestrant (*p* = 0.0215, Day 15) (Fig 5M). Mice injected with GFP cells were treated until Day 32 at which point E2+fulvestrant significantly inhibited tumor size compared to placebo (*p* = 0.0002). No other comparisons reached statistical significance.

**Figure 6:**
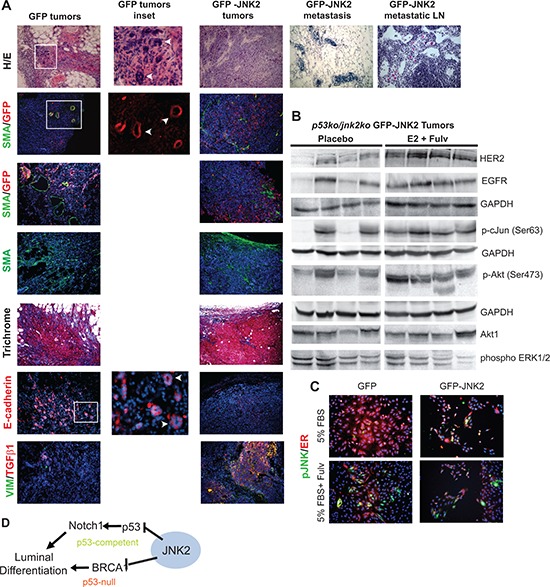
Histologic and western blot analysis of GFP and GFP-JNK2 tumors **A.** Tumors or contralateral glands were fixed and paraffin embedded and later stained with H/E or Masson's trichrome or probed with GFP, SMA or e-cadherin primary antibodies. Cell nuclei were co-stained with DAPI (blue) when probed with SMA, GFP or e-cadherin primary antibodies; **B.** GFP-JNK2 tumor lysates were subjected to western blot analysis and probed with indicated antibodies; **C.** GFP and GFP-JNK2 cells were cultured in 5% FBS with or without fulvestrant (Fulv) for 48 hours. p-JNK (green) and ER (red) were detected using immunocytochemistry; **D.** Proposed model of the mechanism by which JNK2 inhibits luminal commitment of mammary epithelial cells.

Tumors were harvested when reaching target size or on Day 32 and evaluated histologically using Hematoxylin/Eosin (H/E) stain. The GFP-JNK2 tumor cells constituted the majority of the large masses. Limited SMA^+^ myofibroblasts and collagen (by Masson's Trichrome stain) were located at the tumors’ edge (Fig 6A). Within the tumor masses, abundant populations of vimentin and Transforming Growth Factor β1 (TGFβ1) positive tumor cells can be visualized. H/E staining of contralateral glands confirms tumor cells in the glands and lymph nodes. Notably, lymph node localized GFP-JNK2 tumor cells in the E2+fulvestrant treatment group show classical signs of signet-ring cells where abundant intracellular mucin pushes the nucleus asymmetrically [35].

Only limited studies were completed on the GFP masses due to their small size. GFP related masses possess small clusters of GFP^+^ cells (Fig 6A). These cells are enmeshed within SMA^+^ myofibroblasts. In contrast to GFP-JNK2 tumors, GFP tumor cells are e-cadherin positive and organize in pseudo-ductal patterns. Only a few cells stain positive for either vimentin or TGFβ1. These data indicate that while GFP cells activate fibroblasts and collagen deposition, an effect frequently associated with tumor development, they remain indolent or stationary for the remainder of the experiment.

Due to extensive fibrosis, the GFP-related masses were resistant to homogenizing for protein isolation. Conversely, GFP-JNK2 tumors easily disaggregated and potential treatment related changes were evaluated between the placebo and E2+fulvestrant treatment groups, which differed significantly in their growth rates. Fulvestrant stimulated growth has been previously attributed to elevated expression of ErbB family proteins [36]. Higher ErbB2 expression is observed, and to a lesser extent EGFR expression, in the E2+fulvestrant tumors compared to placebo in our model (Fig 6B). No differences in ErbB3 were seen (data not shown). Other notable downstream changes include higher pAkt and p-cJun in the E2+fulvestrant group compared to placebo. Elevated Akt phosphorylation and JNK mediated phosphorylation of cJun may result from higher RTK expression/activity or, alternatively, result from RTK independent fashion since pERK remains unaffected. Through either mechanism, increased phosphorylated cJun suggests that fulvestrant+E2 induces JNK2 activity which, in turn, further promotes tumor progression. To more clearly assess whether fulvestrant induces ER degradation and/or JNK phosphorylation, cell lines were cultured in 5% FBS with or without fulvestrant 10^−7^M for 48 hrs. ER staining (in red) is reduced in fulvestrant treated cells as well as the abundance of pJNK (in green) in both cell lines (Fig 6C). These data further support that fulvestrant degrades ER protein but induces JNK activity in the GFP cells with pJNK signal likely originating from JNK1 isoforms.

Collectively, these data strongly validate a role for JNK2 in the suppression of luminal cell populations (Fig 6D). In the presence of p53, JNK2 inhibits *Notch1* expression, which reduces luminal cell populations. Alternatively, JNK2 inhibits luminal commitment in the absence of p53 by reducing *Brca1* expression and promoting EMT associated tumor growth and progression.

## DISCUSSION

Herein, we address the specific role of *jnk2* in the mouse mammary gland and mammary tumors since previous work showed that high JNK2 tumor expression is associated with poorer disease free survival in patients with basal-like breast cancers [17]. JNK2 limits luminal cell populations in normal mammary and tumor cells, irrespective of p53 status. These findings support that JNK2 mediates EMT and promotes tumor progression. Major efforts are underway to characterize mammary stem and progenitor cell populations to ultimately identify pathways involved in tumor development, recurrence and progression. JNK2 may prove to be one of the more global and easily targeted mediators identified thus far.

Notch1, p53, BRCA1, and ER play integral roles in normal mammary and tumor cell differentiation. Notch1 promotes differentiation but requires temporal regulation of other co-factors such as GATA-3 and BRCA1 to restrict and complete mammary luminal cell differentiation. In a biphasic nature, Notch1 stimulates stem cell differentiation and progenitor expansion. This may explain why *Notch1* is preferentially up-regulated in some breast tumor models [7] where it may expand tumor cells lacking expression of more terminal differentiation genes [37]. A dose-dependent phenotype of Notch1^ICD^ occurs in mammary cells grown in a 3D culture [38]. The higher levels of Notch1^ICD^ expression are associated with small and abortive cultures, whereas lower levels of Notch1^ICD^ are associated with large, hyperproliferative cultures. Our *jnk2ko* phenotype is reminiscent of the latter response, but high Notch1^ICD^ is temporary and primarily localized in TEBs in normal mammary gland. This pattern of expression is consistent with the precocious pubertal phenotype in *jnk2ko* glands without a propensity to develop tumors. By contrast, models generating mammary-targeted supraphysiologic expression of activated Notch1display tumorigenesis through overgrowth of luminal progenitors [39, 40]. Since JNK2 protein is present in both basal and luminal lineages, it could suppress *Notch1* in both stem and progenitor type tumor cells. In our MT model, *Notch1* transcription is elevated in *jnk2ko* tumors in concordance with elevated portions of CK8/18^+^ tumor cells, suggesting a stronger influence on luminal cell populations. A larger luminal target population in *jnk2ko* glands may augment tumor multiplicity resulting from oncogenic stimuli [3]. On the other hand, the lower proliferative index of MT;*jnk2ko* tumors may result from higher p53 expression, replicative stress or reduced cJun activity [23]. These data point to JNK2 conveying positive or negative effects on tumorigenesis that are context dependent. These effects should be considered for strategic therapeutic targeting.

In the *p53ko* model, expanded luminal cell populations are also observed in the absence of *jnk2*, most notably by JNK2 suppression of *Brca1* expression, rather than *Notch1*. Alterations in *Brca1* expression are not detected in the MT/*jnk2ko* tumors, potentially due to wt p53 inhibition of *Brca1* expression [41, 42]. Thus, by using the *p53ko* model, the ability of JNK2 to constrain *Brca1* expression and BRCA1-dependent luminal commitment became evident. BRCA1 is a tumor suppressor that regulates DNA damage response. Microarray analysis further identified increased expression of several other DNA damage related genes in *p53ko;jnk2ko* tumors. More rapid tumor growth was observed in *p53ko;jnk2ko* tumors and cell lines grown *in vitro* and coincided with lower expression of EMT markers and higher luminal marker expression. Several groups have noted a correlation between BRCA1 expression and luminal differentiation [1, 3, 9, 15, 16]. The ability of BRCA1 to mediate mammary tumor differentiation has been attributed to its inhibition of Slug (*Snai2*) and Twist expression, key mediators of EMT [1] [43]. Others have reported that BRCA1 promotes differentiation by de-repressing Polycomb-repressive complex 2 (PRC2) effects on differentiation genes [11]. Thus, inhibition of BRCA1 may be a mode by which JNK2 counters mammary cell differentiation. Whether these BRCA1 functions are related to its role in DNA damage is unclear.

In conditionally deleted *jnk1ko*^Δ/Δ^ *jnk2ko* mammary glands, others have also reported higher proliferation and increased branching. Transplanted *jnk1ko^Δ/Δ^*;jnk2ko** glands also generated more tumors when conditionally expressing *K-Ras*^G12D^ and *p53^Δ/Δ^*. *Jnk* deficient tumors were basal-like, based on limited lineage markers [44]. Implications of these findings are unclear since mutant K-Ras is uncommon in human breast cancer. This group also studied tumorigenesis in systemic *p53ko;jnk1ko* and *p53ko;jnk2ko* mice, but no significant differences were observed in tumor-free survival between control and *jnk-*deficient mice. While *p53^+/−^*; *jnk2ko* mice experienced shorter tumor latencies and tumor-free survival associated with any tumor type [26]. These results bring into question the specific roles of the different JNK isoforms in various tissues. We observed no change in tumor latency in our models with *jnk2* deficiency, but our approach focused solely on mammary tumors by transplanting *jnk2ko* or *jnk2wt* mammary tissue into wildtype hosts.

Given the high frequency of p53 inactivation in human tumors, our *p53ko;jnk2ko* mammary tumor model may prove clinically applicable for understanding mammary tumor biology. It reflects the ability of JNK2 alone to regulate a wide spectrum of tumor cell differentiation states. Namely, JNK2 enhances EMT/stemness, and conveys an ER unresponsive phenotype. The ability of JNK2 to significantly increase the CD24^−^ tumor initiating cell population of a *p53ko;jnk2ko* cell line is also consistent with its differential regulation of BRCA1/ER and EMT. Further characterization of the *p53ko;jnk2ko* cells through limiting dilution assay showed that JNK2 induces tumor initiating cell populations which generate tumors that metastasize, in contrast to GFP masses that failed to progress.

To our knowledge, we are the first to report that JNK2 inhibits ER expression. To better appreciate the potential translational impact of these JNK2 associated changes, we tested whether E2 enhances growth of *p53ko;jnk2ko* GFP or GFP-JNK2 tumors which could then be antagonized by fulvestrant. Unexpectedly, growth rates of GFP and GFP-JNK2 tumors starkly diverged where GFP-JNK2 expression accelerated tumor growth. Histologically, GFP cells cultivated a fibrotic microenvironment in the mammary fat pad, as expected, but tumor cells remained indolent (similar to the limiting dilution experiment), suggesting they are unresponsive to the activated microenvironment. This insufficiency may arise due to their more differentiated phenotype and a low proportion of tumor initiating cells compared to GFP-JNK2 expressing cells. Alternatively, tumor cells may require JNK2 activity to respond to critical integrin and RTK mediated cues from their microenvironment [45].

With regard to the influence of JNK2 on hormone responsiveness, GFP masses were significantly smaller with E2+fulvestrant treatment compared to placebo (*p* < 0.0002) by Day 32, but none of these tumors grew to target size, making this finding of debatable impact. More notably, GFP-JNK2 tumors grew significantly faster with E2+fulvestrant treatment compared to their placebo control, consistent with others showing elevated JNK phosphorylation and high AP1 (Activator Protein-1) transcriptional activity with endocrine resistance [46–48]. AP1 interacts with ER to alter estradiol-mediated responses. AP1 also induces proliferation in response to a variety of stimuli including E2 [49]. In our model, *JNK2* expression prevents fulvestrant associated ER degradation perhaps limiting its efficacy. E2+fulvestrant treatment also increased phosphorylation of cJun on its JNK sensitive residue, indicating that fulvestrant+E2 may directly stimulate JNK to promote tumor progression. Alternatively, higher ErbB2 expression was also noted, a finding reported in other fulvestrant stimulated models [36], and may be the source of elevated p-cJun. Interestingly, cJun can induce ErbB2 expression in association with other factors [50]. Thus, elevated ErbB2 expression and JNK2 activity in response to E2+fulvestrant may be closely interconnected.

Importantly, results obtained from our tumor cell lines somewhat diverge from our spontaneous tumor model from which these cells were derived in that *p53ko;jnk2ko* tumors grew more quickly. One must consider that the cell line is cultured *in vitro* which significantly lessens heterogeneity of the original tumor and removes the microenvironment related influence. Also, the *p53ko* mammary tumor model is heterogeneous in nature, and it is unlikely that any one cell line will accurately reflect all aspect of this model. Reconstituting JNK2 expression in a *p53ko;jnk2ko* cell line provides an opportunity to study JNK2 specific responses while minimizing unaccounted variables associated with the spontaneous tumor model.

Further work is needed to confirm whether the functions attributed to JNK2 herein are dependent on its kinase activity. If this is the case, then clinical evaluation of JNK inhibitors for breast cancer treatment is justified. JNK2 is an attractive target for differentiation therapy, to inhibit metastasis or for endocrine resistant tumors. Newer compounds that covalently bind to JNK offer an improved specificity over previous drugs [51]. We are currently testing their efficacy in various breast cancer models.

## MATERIALS AND METHODS

### Mice

All mouse experiments were performed in accordance with institutional and national guidelines and regulations. Animal procedures and experiments are pre-approved by the IACUC at the University of Texas, Austin. Balb/c *p53ko* mice were obtained from Dr. Dan Medina (Baylor College, Houston, TX) and *jnk2ko* mice from Dr. Lynn Heasley (University of Colorado, Aurora, CO) [52]. MT transgenic mice were obtained from the NCI Mouse Consortium [53]. MT and *jnk2ko* mice were backcrossed >10 generations to Balb/c background as described [23].

### Reagents

Unless otherwise stated, chemical reagents were purchased from Sigma. Antibodies used include Six1 (Origene), p63 (Millipore), Ki67 (Neomarkers), SMA (DakoCytomation), Notch1 (Millipore). Antibodies obtained from EBioscience are CD24-PECy7, CD49f-PE. Cell Signaling antibodies include MMP9, Cleaved Caspase-3, Notch1^ICD^, E-cadherin, GAPDH, Vimentin, Lef-1, and Zeb1. Santa Cruz antibodies include BRCA1, CK8/18, ER, PR, mouse HRP-conjugated secondary antibody, rabbit HRP-conjugated secondary antibody. Pharmingen antibodies include the mouse lineage panel kit and BrdU-FITC kit, used per manufacturer's instructions. Adenoviral MAM51 was a kind gift from Karine LeFort and G. Paolo Dotto [20]. Notch1 promoter constructs were a kind gift from Dr. Takashi Yugawa [24]. BRCA1 promoter constructs were a kind gift from Dr. Robert Glazer [54]. Fulvestrant and placebo control were a generous gift of Astra Zeneca (Cheshire, UK).

### Whole mounts

Number four glands from virgin mice were fixed in Carnoy's fixative. Glands were stained in carmine alum and mounted with Permount (Fisher Scientific). Slides were imaged using an Olympus MVX10 microscope and Image Pro Plus software (Media Cybernetics). Ductal extension was scored as the average distance of the five furthest traversed TEBs from a line drawn through the center of the lymph node, perpendicular to the direction of extension. Branching and TEBs were quantified by counting individual structures per gland.

### Tissue processing and histology

Number four glands from virgin mice were fixed and paraffin embedded. Five micron sections were treated with Citrisolv (Fisher Scientific), rehydrated and boiled in 10mM Na Citrate. Tissue was permeabilized and treated with H_2_O_2_ before primary antibody incubation. Tissues were then treated according to ABC kit (Vector Labs) and DAB substrate kit (Abcam) directions and then mounted with VectaMount (Vector Labs). Tissues for immunofluorescence (IF) were incubated with fluorescently-labeled secondary antibodies and mounted with VectaShield with DAPI (Abcam). Images were obtained using an Olympus CKX41 bright-field microscope with QCapture Pro software (Media Cybernetics). IF images were obtained on an Eclipse TE200 microscope (Nikon) using Image Pro Plus Software.

### Primary mammary epithelial cell culture and imaging

Primary Mammary epithelial cells were isolated from 8–10 wk, virgin glands. Briefly, glands were minced and treated with collagenase. Mammary epithelial cell organoids were isolated by differential centrifugation and disaggregated with 0.025% trypsin/EDTA (Invitrogen). After filtration, mammary epithelial cells were grown on Matrigel (BD Biosciences) according to established protocols [55] [56]. Acinar diameters were measured from bright field images with QCapture Pro Software. IF images were obtained using an SP2 AOBS Confocal Microscope (Leica) as previously described [56].

### Cell lines and BRCA1 and GFP-JNK2 expression

MT/*jnk2ko* and *p53ko;jnk2ko* cell lines were derived from mammary tumors [23]. Cells were maintained in DMEM/F12 (1:1) medium (Mediatech Inc) supplemented with 10% FBS (Gemini), 10 ug/ml insulin (Lilly), 5 ng/ml EGF (Peprotech), penicillin, and streptomycin (Life Technologies). The BRCA1 expression plasmid (Dr. Elledge) obtained through Addgene [57]. GFP-JNK2 expression was performed as previously described [23].

### Western blot and MTT assay

Assays were performed as published [17].

### qPCR

RNA was isolated from mammary epithelial cells in 3D culture using Trizol Reagent (Invitrogen). Primers were designed using PerlPrimer. Expression was measured by the SYBR Green method in a Stratagene M × 3005 p qPCR Machine (Agilent Technologies). Relative expression was calculated using standard curves with MxPro Software (Agilent Technologies). All assays were performed in triplicate of each sample. In tumor studies, each individual tumor RNA sample was analyzed in triplicate and the means were grouped to assess overall gene expression effects.

### Luciferase assay

Subconfluent MT/*jnk2ko* GFP and GFP-JNK cells were transfected with N1PR, N1PRp53mut, or N1PRless luciferase constructs and CMV-Beta-galactosidase. Cells were harvested using Reporter Lysis Buffer (Promega). Samples were read on a Synergy 4 Plate reader (BioTek).

### p53ko mammary gland transplants

Mammary glands from sexually mature mice were transplanted into cleared fat pads of 3 wk wildtype females. Two weeks after transplantation, recipients were implanted with pituitary isografts within the kidney as described [25]. Glands were palpated until tumors formed and reached target size of 1.5 cm diameter.

### Flow cytometry

Single cell suspensions were isolated from mammary glands as above or from culture and incubated with antibodies as recommended by manufacturer. Labeled cells were run on Millipore Guava Easy Cyte 8HT or BD FACSAria II and analyzed using FloJo 9.3.1 software (Treestar Inc). All assays were performed in triplicate.

### Microarray analysis

Total RNA was collected, purified, and hybridized to Agilent custom 4 × 180 K microarrays as previously described [58]. The microarray data were uploaded to the University of North Carolina Microarray Database (UMD) (https://genome.unc.edu/pubsup/breastGEO/clinicalData.shtml) and to the Gene Expression Omnibus (GEO) under accession number GSE40226. Hierarchical clustering was performed using Gene Cluster 3.0 [59] and the data viewed using Java Treeview version 1.1.5r2 [60]. Statistically significant expression changes between tumor genotypes were determined using a 2-class unpaired significance analysis of microarray (SAM) analysis, with genes having a false discovery rate (FDR) ≤ 5% considered statistically different.

### BrdU assay

Subconfluent cells were incubated with BrdU for one hour and then stained with antibodies per manufacturer's instructions.

### Limiting dilution tumor assay

Cell lines were trypsinized and recovered in full media, passed through a 40 um cell strainer and counted. Serial dilutions of 10^4^, 10^3^, and 10^2^ cells in 50 uL were sterile filtered in normal saline (Biochemika). 50 uL of cells were injected into #2 mammary glands of nude mice using a Hamilton syringe. Tumors were palpated thrice weekly. L-Calc v1.1 (Stemsoft Software) was used to determine tumor initiating cell frequency.

### Chromatin immunoprecipitation assay

Cells were fixed in 1% formaldehyde. Cross-linking reactions were stopped and centrifuged cells were then lysed and chromatin was sheared by bath sonication. Samples were then centrifuged and chromatin-containing supernate was divided for input, IgG negative control (Calbiochem), and p53 (Cell Signaling) immunoprecipitation (IP). Chromatin was IP'ed with protein A sepharose beads (Fisher Scientific) and pre-cleared with sonicated salmon sperm DNA (Amresco). Samples were washed with progressively higher salt solutions as previously described [61], and eluted from beads. Cross-links were reversed, and chromatin precipitated. After re-suspension, chromatin was treated with Proteinase K (Fisher Scientific). After digestion, DNA was isolated using a DNEasy kit (Qiagen) according to manufacturer's instructions. Samples were amplified 30 cycles by PCR using specific primers, listed in Supplemental procedures.

### Orthotopic tumor growth experiments

Female ovariectomized athymic mice were implanted s.c. with E2 pellets (0.72 mg, 42-day release; Innovative Research of America). The next day, 2 × 10^6^ *p53ko/jnk2ko* GFP or GFP-JNK2 cells suspended in sterile PBS were injected into the number 4 mammary gland. When tumors became palpable, mice were injected subcutaneously with fulvestrant (5 mg) or placebo weekly [62]. Tumor volumes (volume = width^2^ × length/2) were measured thrice weekly and harvested ≥ 750 mm^3^. Tumors were flash-frozen in liquid nitrogen or fixed in 10% formalin prior to paraffin-embedding. Frozen tumors were homogenized in lysis buffer and subjected to SDS-PAGE, transferred to nitrocellulose and analyzed by immunoblot analysis.

### Statistics

Statistical analyses were performed using Prism Software (GraphPad). A nonparametric, two-tailed *t*-test was employed to determine significance between two groups. Significance for survival data was determined using Log-rank test, where *p* < 0.05 was considered significant. The 1-way ANOVA was employed to determine significance amongst multiple groups followed by post-hoc *t*-test to compare two samples. Error bars are shown as standard error of the mean. Significant data from *t*-tests are indicated on figures as follows: *p < 0.05, **p < 0.01, ***, *p* < 0.0001.

## SUPPLEMENTARY DATA


