# Supplementary material for: c-Jun N-terminal kinase 2 prevents luminal cell commitment in normal mammary glands and tumors by inhibiting p53/Notch1 and breast cancer gene 1 expression
Source: Oncotarget. 2015 Apr 25;6(14):11863–81. doi: 10.18632/oncotarget.3787 (PMC4494910; doi:10.18632/oncotarget.3787)
Supplement: Supplementary file 1 [file oncotarget-06-11863-s001.pdf]

SUPPLEMENTARY DATA

Supplemental Experimental Procedures:
Primers used (all are against mouse, unless otherwise indicated):
qPCR primers:
hes1 NM_008235.2 (5′-CACAGAAAGTCATCAAAGCC and 5′-TGCTTGACAGTCATTTCCAG),
notch1 NM_008714.3 (5′-CTACAGAAGGTTACACAG and 5′-CAGAGGTAGGAGTTGTCACG),
p53 NM_001127233.1 (5′-TGAACCGCCGACCTATCCTTA and GGCACAAACACGAACCTCAAA),
twist1 NM_011658.2 (5′-AATTCACAAGAATCAGGGCGTGGG and 5′-TCTATCAGAATGCAGAGGTGTGGG),
snai1 NM_011427.2 (5′-TCCAAACCCACTCGGATGTGAAGA and 5′-TTGGTGCTTGTGGAGCAAGGACAT),
snai2 NM_011415.2 (5′-CACATTCGAACCCACACATTGGCT and 5′-TGTGCCCTCAGGTTTGATCTGTCT),
zeb1 NM_011546.3 (5′-CAGTGTTCCATGTTTAAGAGCA and 5′-GTCTTTCATCCTGGTTTCCG),
klf4 NM_010637.3 (5′-CATTATCAAGAGTCTATGCCA and 5′-CACAGTGGTAAGGTTTCTCG),
cdh1 NM_009864.2 (5′-GCCAAGTACATCCTCTATTCTC and 5′-GCAACGAATCCCTCAAAGAC),
gapdh NM_008084.2 (5′-CGTGGAGTCTACTGGCGTCTTCAC and 5′-CGGGGATGATGAGCCTTTTGGC),
brca1 NM_009764.3 (5′-CCAAAGAAGTAATGACCGTG and 5′-GCTAACTATCCACTTTCCTCC),
*gata-3*NM_008091.3 (5′-ACGAATCCAGCACAGAAGG and 5′-ATGTCCCTGCTCTCCTTG),
ck14 NM_016958.1 (5′-TCTTCAGCAAGACAGAGGAG and 5′-CTCCAGGTTATTCTCCAGGG),
p63 NM_001127259.1 (5′-GTTCAATGAGGGACAGATTGC and 5′-GAATTCAGTGCCAACCTGTG),
Human *brca1* NM_007294.3 (5′-CCCAGAAGAATTTATGCTCGT and 5′-CATTGACCACATCTCCTCTG),
Human *gapdh*BC096440.1 *(*5′-AAGGTGAAGGTCGGAGTCAA and 5′-AATGAAGGGGTCATTGATGG).
ChIP primers:
p21 NC_000006.11 (5′-GATTTCCTTTCTATCAGCCC and GTCACAAGATACATACCACCT),
gapdh NC_000012.11 (5′-GCCAAAGACAGAAGCCAGGA and 5′-CAGGATAGGACTCAGGGAATACAG),
notch1 NC_000068.7 (5′-GTGACCGTGGAACGTCTA 5′-CTGTCCTAGGGCTCCAC)

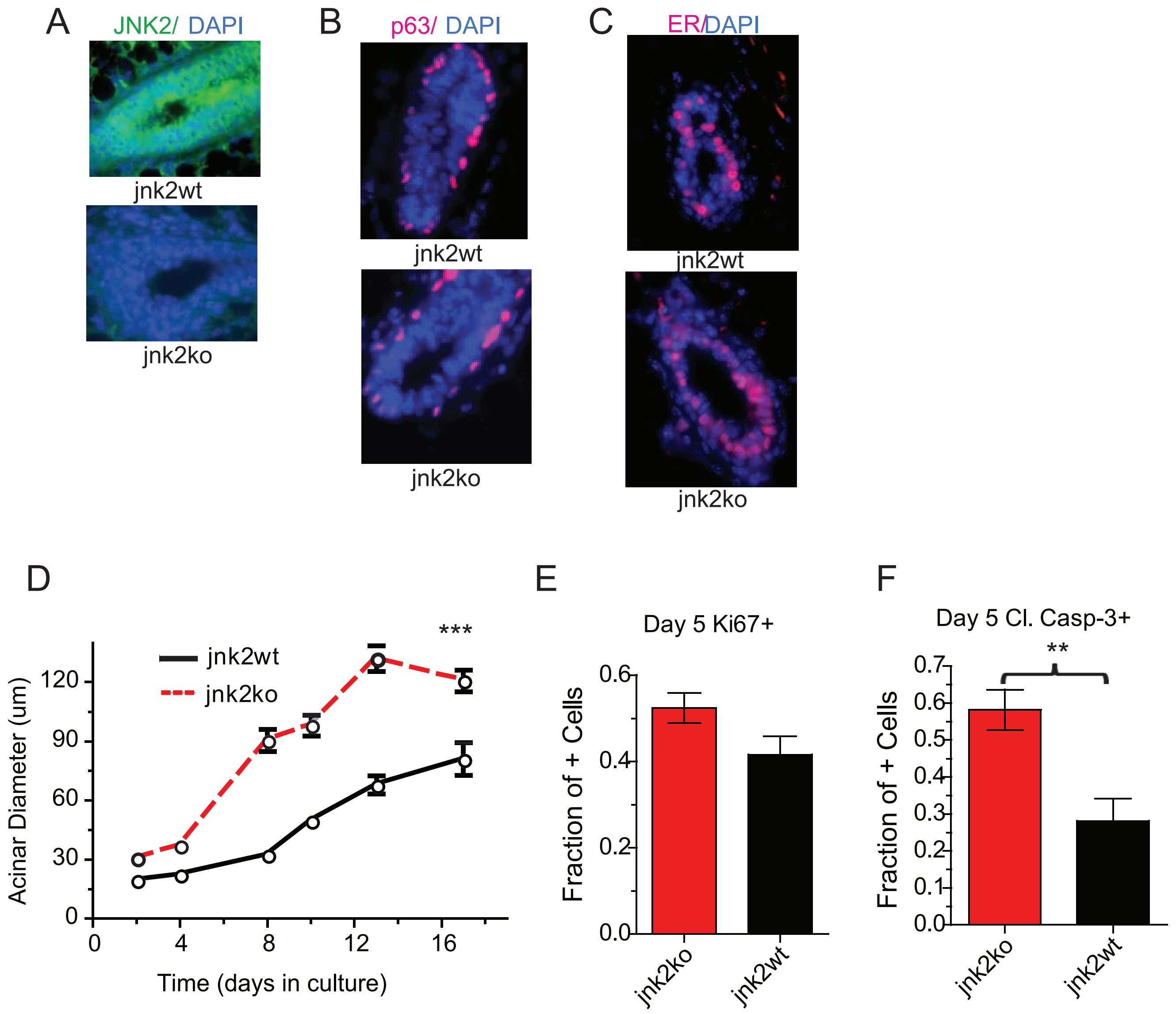

Supplementary Figure S1: Effect of JNK2 on mammary epithelial cells. A. Representative images of adult (8 wk-old) ducts probed for JNK2 expression; **B-C.** Images of p63^{+} basal cells and ER^{+} luminal cells in adult ducts ($n = 3$); **D.** *Jnk2wt* and *jnk2ko* mammary epithelial cells were isolated and grown in 3D culture. Acinar diameter was measured at indicated time points. Linear regression analysis was performed, ***$p < 0.0001$; **E-F.** Quantification of Ki67^{+} and cleaved caspase 3^{+} cells grown in 3D culture. Nonparametric t-test was performed, **$p < 0.001$.

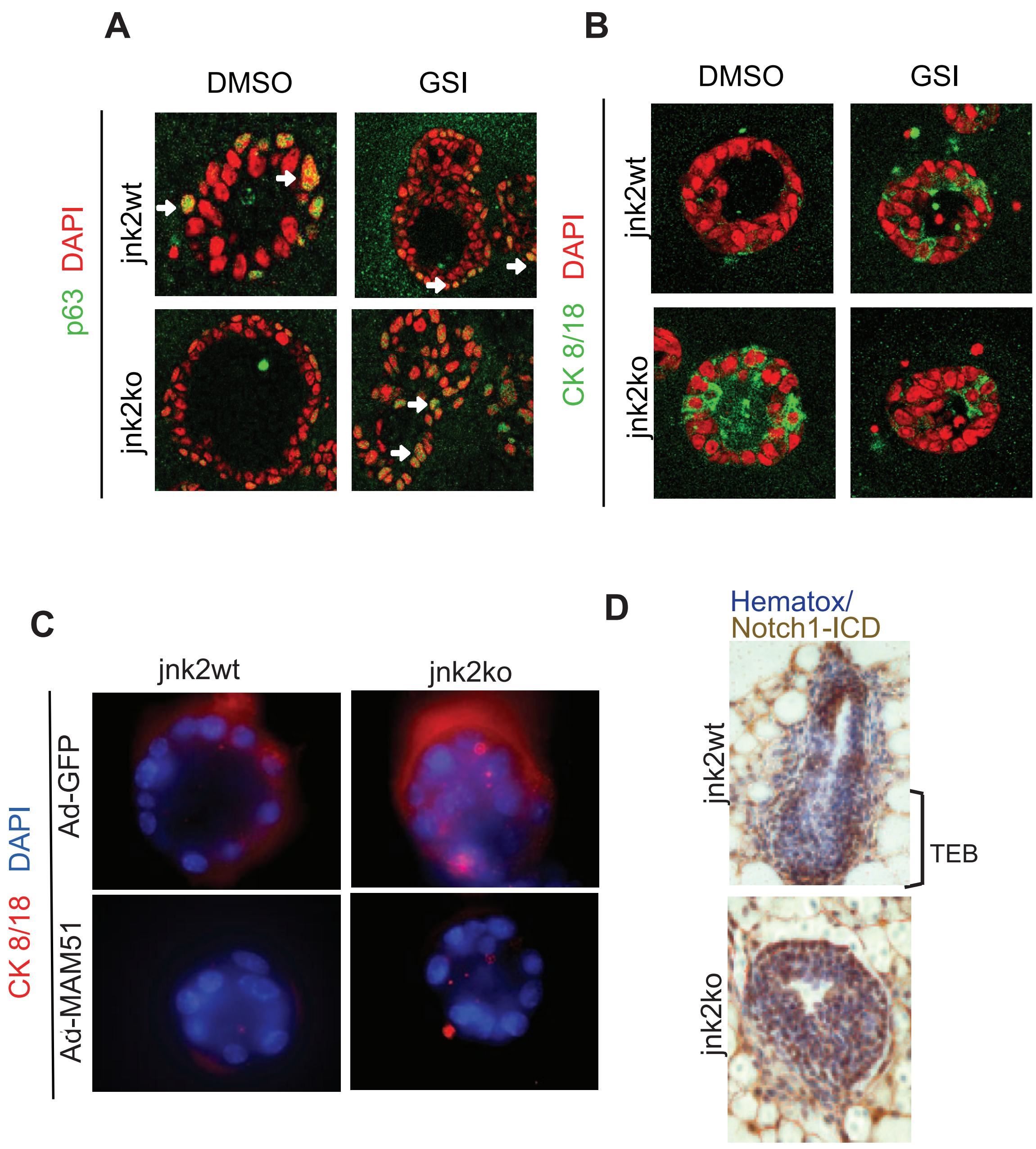

Supplementary Figure S2: Representative images of JNK2 effects on mammary cell differentiation and Notch activity. A. Representative staining where arrows indicate p63+ cells in 3D culture; **B-C.** Representative staining of ER^+ mammary cells in 3D culture; **D**. Representative staining of $Notch1^{ICD}$ in TEBs.

A **B**

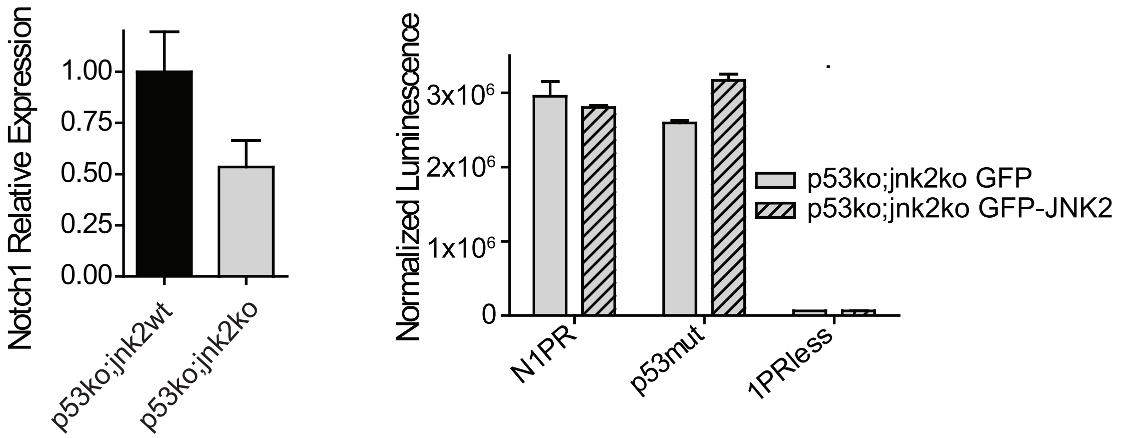

C

BioCarta	NOM p-val	FDR q-val
ATRBRCAPATHWAY	<0.0001	<0.0001
ATMPATHWAY	<0.0001	0.0028

D

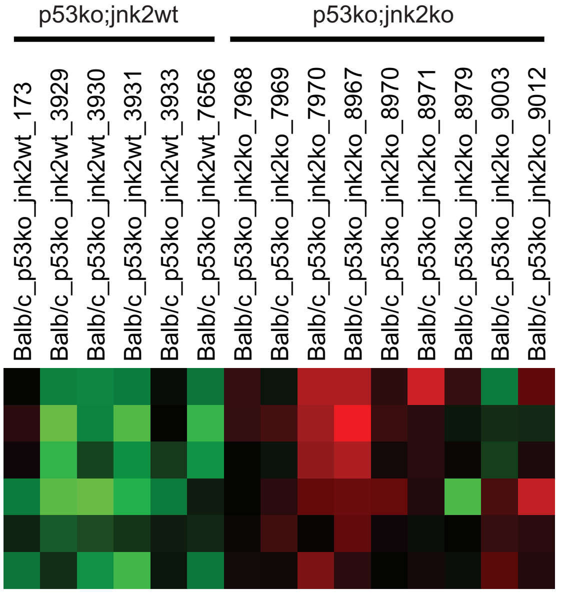

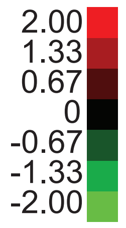

Probe ID	Gene	Fold Change	%FDR
A_52_P117090	Chek2	1.93	4.38
A_52_P18267	Brca1	2.16	4.38
A_52_P556462	Fancd2	1.76	4.38
A_51_P357182	Mapk8	2.36	2.40
A_52_P574881	Hus1	1.42	4.38
A_52_P369581	Atm	1.83	2.06

Supplementary Figure S3: JNK2 effects on gene expression in *p53ko* tumors or tumor cell lines. A. RNA from tumors was analyzed for Notch1 expression using qPCR. A nonparametric, two-tailed *t*-test was used to detect statistical differences between two groups; **B.** *p53ko;jnk2ko* GFP and GFP-JNK2 expressing cells were transfected with Notch1 promoter-luciferase constructs and analyzed for promoter activity. A 1 Way ANOVA with post-hoc *t*-test was performed; **C.** Gene Set Enrichment Analysis (GSEA) of *p53ko;jnk2ko* and *p53ko;jnk2wt* tumor microarrays shows upregulation of the ATRBRCAPATHWAY and ATMPATHWAY gene sets in *jnk2ko* tumors; **D.** Genes significantly elevated with false discovery rate (FDR) of < 5% in the ATM/BRCA1 pathway.

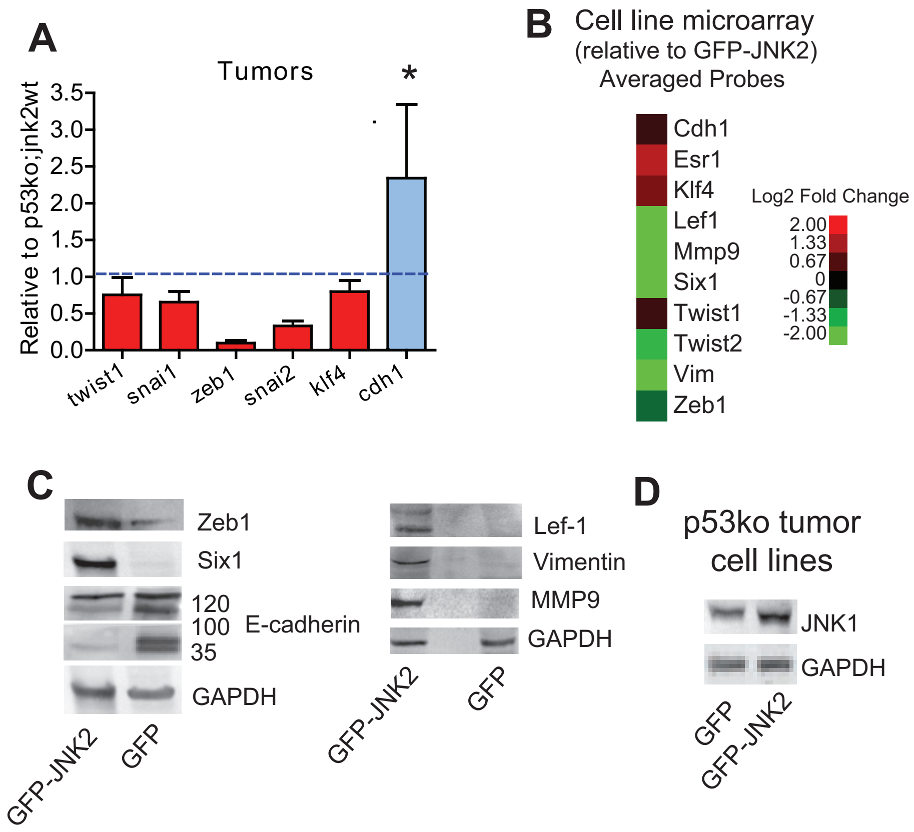

Supplementary Figure S4: JNK2 promotes EMT in the p53ko tumor model. A. Expression of mesenchymal/stem (red) and epithelial (blue) markers in *p53ko* tumors (n = 8), **B.** Fold changes observed in microarray analysis of EMT/stem and differentiation markers in *p53ko;jnk2ko* GFP and GFP-JNK2 cell lines; **C.** Expression of EMT/stem-related proteins in *p53ko;jnk2ko* GFP and GFP-JNK2 cell lines by western blot; **D.** Expression of JNK1 in *p53ko;jnk2ko* GFP and GFP-JNK2 cell lines by western blot. A nonparametric, two-tailed t-test was also performed for A. *$p < 0.05$.

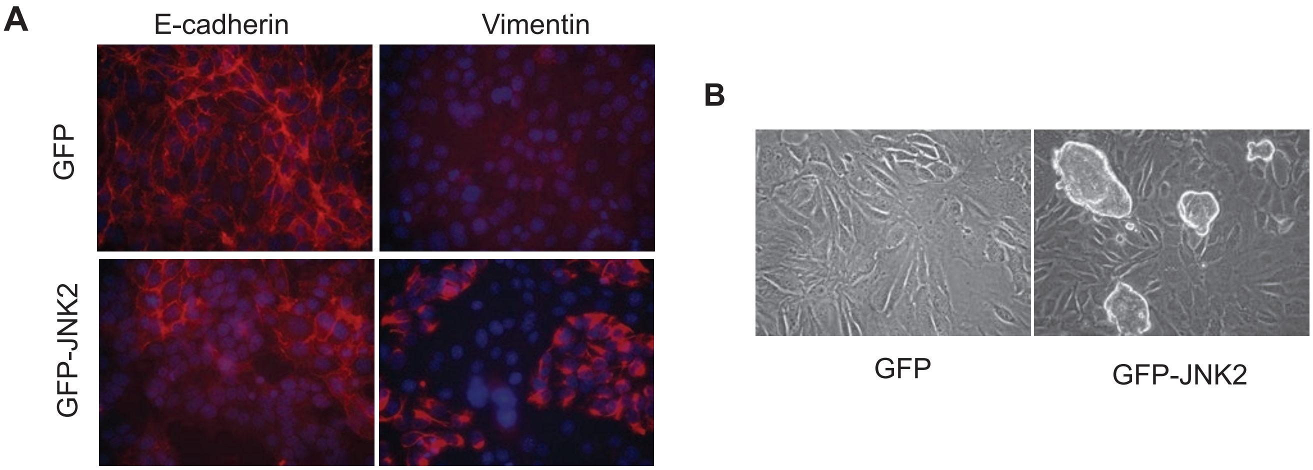

Supplementary Figure S5: JNK2 promotes vimentin expression and inhibits e-cadherin in the *p53ko;jnk2ko* cell line. **A.** Adherent cells were stained using vimentin and e-cadherin primary antibodies; **B.** Adherent cells grown in culture.
